# Survival of a patient with multiple-recurrent giant retroperitoneal dedifferentiated liposarcoma for 15 years: A case report

**DOI:** 10.3389/fsurg.2022.916802

**Published:** 2022-11-07

**Authors:** Hao Xia, Fang Fang, Haijuan Yuan, Yimei Tu

**Affiliations:** ^1^ Department of Gastrointestinal Surgery, Northern Jiangsu People’s Hospital, Clinical Medical College, Yangzhou University, Yangzhou, China; ^2^ Department of Hernia Surgery, Northern Jiangsu People’s Hospital, Clinical Medical College, Yangzhou University, Yangzhou, China

**Keywords:** giant retroperitoneal liposarcoma, recurrence, dedifferentiated, surgery, prognosis, computed tomography, target genomic therapy

## Abstract

**Background:**

Retroperitoneal liposarcoma (RPLS) is a variety of soft tissue sarcoma that originates from mesenchymal cells. A tumor measuring greater than 30 cm is called a “giant liposarcoma.” A part of the neoplasm tends to grow in size, recur locally, or metastasize distantly. In those with such a condition, long-term survival is uncommon. Therefore, it is necessary to present a uniform and optimized program to improve the prognosis.

**Methods:**

By successfully treating a multiple-recurrent giant retroperitoneal dedifferentiated liposarcoma (RP DDLPS) in July 2010, we hope to devise more comprehensive strategies to improve diagnosis, therapy, and outcome.

**Results:**

In July 2010, we thoroughly resected a giant multifocal RPLS with a concomitant part of the gastric wall. The histopathological examination revealed a high-grade (grade III) dedifferentiated liposarcoma. The patient was discharged uneventfully on the 15th postoperative day. She relapsed after 16 months and needed another complete excision. After 9 months, she died after the fourth recidive. The patient had experienced four recurrences and underwent operations with 15 years of follow-up.

**Conclusions:**

The above demonstrates that we were able to successfully treat the multirecurrent giant RPLS, despite the patient’s poor medical condition, with meticulous management. Moreover, this indicates that long-term survival could be achieved for high-grade RP DDLPS.

## Introduction

Retroperitoneal sarcomas (RPSs) are rare malignancies that develop from mesenchymal tissues. They account for approximately 15% of all sarcomas, with an estimated incidence of 3–4/1,000,000 of the population per year (1). Liposarcoma is the most common RPS and accounts for 41% of all RPS (2). RPLS may frequently occur at any age, with a peak incidence between 40 and 60 years of age, and the distribution is equal between genders (3, 4). As protuberances develop in the vast, expandable retroperitoneum, they often present with atypical symptoms and grow to enormous proportions before being detected. Lewis reported that masses greater than 10 cm account for approximately 60% of all cases (5), while diameters greater than 30 cm are termed “giant liposarcoma” but are rarely diagnosed. RPLS is one of several pathological variants.

Furthermore, they tend to recur locally and distantly, possibly infiltrating adjacent organs or tissues, with the former being the primary cause of death (6, 7). The mainstay of treatment is *en bloc* resection of tumors and contiguous structures. Patients often undergo multiple operations after relapses, but the resection rate tends to decline gradually, leaving few long-term survivors (5). This paper focuses on the diagnosis, treatments, and prognosis of managing a multiple-recurrent giant RP DDLPS. Meanwhile, we also reviewed the literature of 12 cases with a giant RPLS measuring over 30 cm by searching English language articles through the PubMed database. Until now, this case represents the longest duration of follow-up in this variety of cancers reported in the literature in English.

## Case report

### Case description I

In July 2010, a 50-year-old female with a poor medical condition was admitted to our medical center with abdominal distension for 2 months. Her past surgical history included a complete resection of RPLS in 1997 with four courses of postoperative chemotherapy, another thorough excision for the first recurrence in 1999 with no further treatments, and laparoscopic cholecystectomy in 2003, all in other hospitals. Her family and psychosocial history were unremarkable, and she had no known genetic diseases. The physical examination revealed abdominal distension. Meanwhile, a diffuse and tough mass was palpated in the right abdomen with local tenderness and was stable with ill-defined margins. As for laboratory results, hemograms indicated mild anemia (hemoglobin 101 g/L), hepatorenal functions revealed hypoproteinemia (albumin 27 g/L), and tumor antigens showed CA125 185.80 KU/L. The chest radiography demonstrated bilateral pleural effusion and no space-occupying lesions.

**Figure 1:**
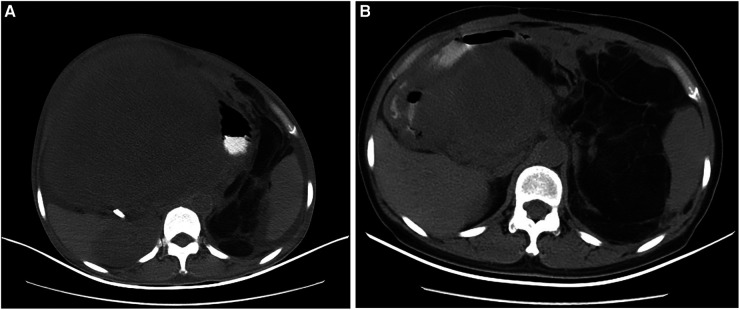
The imaging of CT scanning indicated for the second and third recurrence of the female patient in July 2010 and November 2011, respectively. (**A**) The giant tumors with adipose density occupied almost the entire abdominal cavity (July 2010); (**B**) The tumors originated from the hepatic hilar region and oppressed adjacent organs and tissues (November 2011).

The features of abdominal and pelvic contrast-enhanced computed tomography (CT) included multifocal, giant tumors of roundish shape with adipose density that were suspicious for liposarcoma (Figure 1A); the nodular masses existed in the liver–stomach clearance; the right kidney and liver were both compressed to deformation, and the hepatic cysts were obvious; the left kidney was dislocated to the intraperitoneal cavity, and abundant ascites was observed.

**Figure 2:**
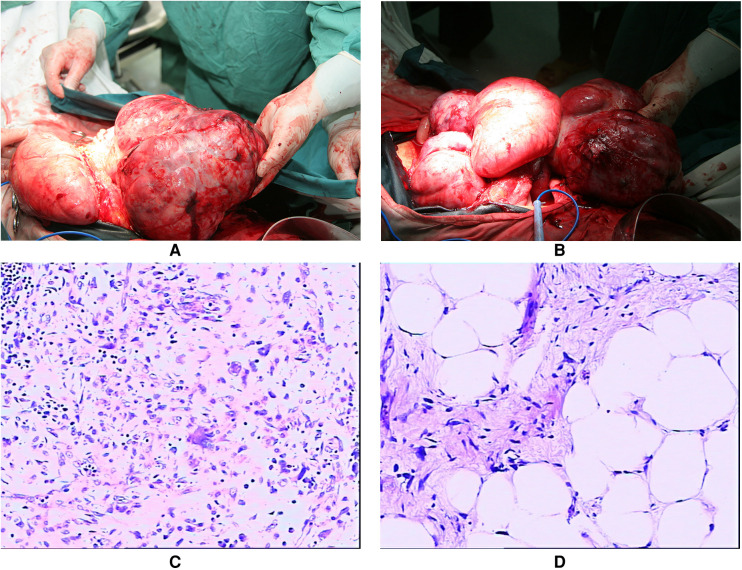
The gross appearance of the totally excised tumors presented in the operation (July 2010). (**A**) The giant tumor shaped like lobulated or dumbbell (measured 45×30×20 cm) with rich blood supply; (**B**) The multifocal tumors were well-encapsulated with macroscopic safe margins. The histopathological presentations of the tumors originated from the retroperitoneum and the tumors infiltrated the stomach (July 2010). (**C**) The retroperitoneal tumors were diagnosed as dedifferentiated liposarcoma; (**D**) Tumors of the stomach were diagnosed as sclerosing liposarcoma, dedifferentiation in focal areas.

After meticulous planning, we performed laparotomy through a right exploratory incision, indicating approximately 800 ml of light hemorrhagic ascites and extensive adherence of the omentum and intestinal loops. A giant tumor originated from the right middle and upper quadrant of the retroperitoneum, which crushed the liver upward to the right and posterior of the subdiaphragm, oppressed the flexura hepatica coli and abdominal wall rightward, pushed the mesocolon transversum downward to 10 cm below the umbilicus, and crushed the hepatogastric and gastrocolic ligaments anterior to the abdominal wall. The left margin of the tumor was adjacent to the hilus lienis. The tumor was shaped like a lobulated dumbbell, measuring 45 cm × 30 cm × 20 cm in size. Another yellow and white tumor in the mesocolon transversum measured 20 cm × 8 cm × 5 cm in diameter. Three small masses linked by the basis pontis of the greater gastric curvature extruded to the hilus lienis. In surgery, we separated gastrohepatic and hepatocolic ligaments, dissociated the capsule of the tumor, and separated the tumor from the adjacent tissues. Due to the enormous size, we had to separate a part of the mesocolon transversum and keep the tumor from metastasizing into it. We discovered that the tumor was hiding behind the transverse colon and the stomach, turned out it, and therefore, we detached the root of its capsule and severed and ligated the feeding veins before completely resecting the giant tumor. All tumors were well-encapsulated and completely removed with a concomitant part of the gastric wall (Figures 2A, B). The specimen weighed 6.65 kg, and the estimated blood loss was 2,800 mL.

The histopathological report indicated that in the retroperitoneal tumors, the oncocytes were heterogeneous and distributed like star networks with loose mesenchyme. Small fatty components were near the capsules in focal areas (diagnosed as dedifferentiated liposarcoma); in the tumors of the gastric wall, the nuclei were heterogeneous with clear cytoplasm. The lip blasts with abundant vessels were in focal areas (diagnosed as sclerosing liposarcoma, dedifferentiation in focal areas) (Figures 2C, D).

The patient was discharged on the 15th postoperative day with an uneventful course. The follow-up was done every 3 months for 16 months, and no signs of recurrence or metastasis were detected.

### Case description II

**Figure 3:**
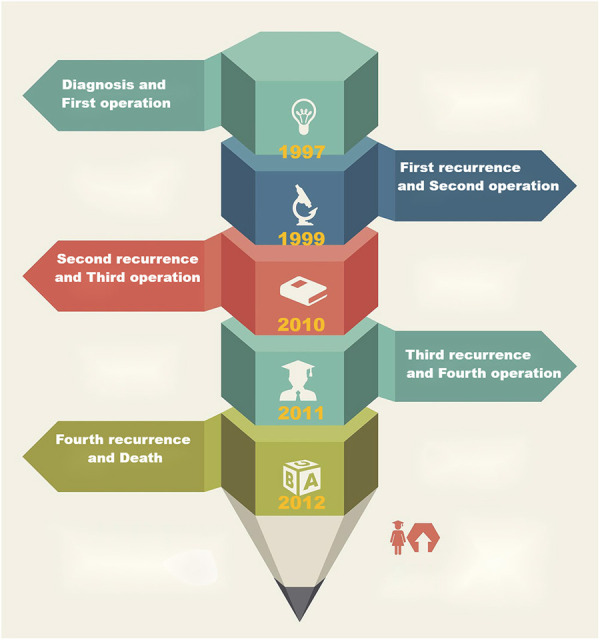
The timeline shows that the patient underwent operation four times and experienced recurrences, respectively, and 15 years of follow-up in all.

The patient was readmitted to our hospital on November 2011. The abdominal and pelvic CT revealed multifocal, class-round tumors originating from the hepatic hilar region and oppressing the adjacent intestinal canal, caudate lobe, inferior vena cava (IVC), and aorta abdominalis (Figure 1B); another mass was located in the middle and lower abdomen; the tumors were all enhanced inhomogeneously; the left kidney was displaced anterior to the intraperitoneal cavity; and a class-round tumor and low density existed in the right ovary but was not enhanced, which was diagnosed as a recurrence of multifocal RPLS. The mass was considered a teratoma of the right ovary. She was submitted for another laparotomy. We resected a mass adjacent to the porta hepatis, a tumor and several small masses in the mesocolon transversum, and a tumor behind the mesosigmoid. The excised tumors measured 15 cm × 10 cm × 6 cm, 10 cm × 6 cm × 5 cm, and 8 cm × 5 cm × 5 cm, respectively. The pathological results confirmed dedifferentiated RPLS. She was discharged without incident but unfortunately died during the 4th recurrence in August 2012.

(The timeline is shown in Figure 3.)

## Literature review

In the literature in the English language published on PubMed from 1982 to December 2021, only 12 cases with giant RPLS greater than 30 cm in diameter have been reported (2, 8–18). Among the 13 patients (including ours), nine were male (69.2%) and four were female (30.8%), with a median age of 64 years (24–82). They mostly complained of vague symptoms such as abdominal discomfort and distension. CT imaging was the main examination for diagnosis, while only two patients (15.4%) received fine-needle aspiration cytology for the preoperative diagnosis. All the 13 patients underwent surgery, and seven of them (53.8%) were combined resections, including six nephrectomies (46.2%), one left colectomy (7.7%), one partial diaphragmatic resection (7.7%), one adrenalectomy (7.7%), and one part of gastric wall excision (7.7%). A total of 12 out of 13 cases (92.3%) achieved R0 resection. Furthermore, all 13 patients were discharged uneventfully without any complications. With regard to the histopathological types, six were dedifferentiated (46.2%), five were well-differentiated (38.5%), one was pleomorphic (7.7%), and one was well-differentiated/myxoid (7.7%). Meanwhile, in accordance with the grading system of the French Federation Cancer Centers (FNCLCC), six cases were grade I (46.2%), two cases were grade II (15.3%), and the remaining five cases were grade III (38.5%). In the group of 13 cases, one patient accepted neoadjuvant chemotherapy (7.7%) with no benefit, and one received postoperative radiotherapy (7.7%) but with no further evaluation. The months of follow-up were in the range of 8–181, during which two patients had recurrences at 16 and 21 months. Besides, two longer-surviving patients had local recurrences at 60, 108, and 120 and at 24, 156, 172, and 181 months after initial surgeries, respectively (Table 1).

**Table 1:** Clinical characteristics, managements, histological features, follow-up, and outcomes of 13 patients with a giant RPLS measuring greater than 30 cm.

No.	Author/ Year	Sex/age (years)	Main clinical presentation	Imaging for diagnosis	Preoperative biopsy	Range of resection	Size (cm)	Complications	Pathological type	FNCLCC grade	R0 resection or not	Adjuvant therapy	Follow-up (mo)	Evolution
1	Ianosi 2007 (8)	F/49	Lumbar pain	US, CT	No	Wide excision with right kidney	35 in diameter	No	Dedifferentiated	Grade III	Yes	No	36	Local recurrence at 21 mo postoperation and resection
2	Herrera-Gómez 2008 (2)	M/24	Abdominal pain, constipation, fever and a 20-kg weight loss	CT	Yes (FNAC)	Wide excision	80 × 50 × 35	No	Dedifferentiated	Grade II	Yes	Neoadjuvant chemotherapy	14	No recurrence
3	Han 2010 (9)	M/82	Abdominal mass and associated abdominal discomfort	CT	No	Wide excision with the left kidney and adrenal gland	30 × 30 × 8	No	Well-differentiated	Grade I	Yes	No	18	No recurrence
4	Hashimoto 2010 (10)	M/41	Abdominal swelling, marked leg edema, a 30 kg weight gain, and cough	CT	Yes (FNAC)	Wide excision with the right kidney	45 × 40 × 30	No	Dedifferentiated	Grade III	Yes	No	12	No recurrence
5	Caizzone 2015 (11)	F/64	Progressive volumetric increase of the abdomen	CT	No	Wide excision with the right kidney	42 × 37 × 18	No	Pleomorphic	Grade III	Yes	No	24	No recurrence
6	Oh 2016 (12)	F/71	Progressive abdominal distension	US, CT	No	Wide excision	45 × 30 × 11	No	Well-differentiated/myxoid	Grade I	Yes	No	28	Local recurrence at 16 mo postoperation and resection
7	Zeng 2017 (13)	M/45	Gradual increase in abdominal girth and edema in both lower extremities	CT	No	Wide excision	65 × 45 × 30	No	Well-differentiated	Grade I	Yes	Radiotherapy	8	No recurrence
8	Hazen 2017 (14)	M/64	Abdominal distension	CT	No	Wide excision with left radical nephrectomy and left colectomy	60 × 42 × 31	No	Dedifferentiated	Grade III	Yes	No	N/A	N/A
9	Herzberg 2019 (15)	M/75	Rapid weight loss, reduced appetite, intermittent constipation, and a growing abdominal circumference	US, CT	No	Wide excision with the left kidney and partial diaphragm	35 × 29 × 20.5	No	Dedifferentiated	Grade II	Yes	No	24	No recurrence
10	Xu 2020 (16)	M/65	Increasing abdominal girth	CT	No	Wide excision	37 × 32 × 26.5	No	Well-differentiated	Grade I	Yes	No	12	No recurrence
11	El-Helou 2020 (17)	M/70	Dizziness and fatigue	CT, MRI	No	Wide excision	50 × 30 × 18	No	Well-differentiated	Grade I	Yes	No	120	Local recurrence at 60, 108, and 120 mo from initial diagnosis and respective resection
12	Kuperus 2021 (18)	M/45	Left scrotal swelling and pain	CT, MRI	No	Wide excision	39 in diameter	No	Well-differentiated	Grade I	No (R1)	No	8	No recurrence
13	Our case 2022	F/50	Abdominal distension and masses	CT	No	Wide excision with a part of the gastric wall	45 × 30 × 20	No	Dedifferentiated	Grade III	Yes	No	181	Local recurrence at 24, 156, and 172 months from initial diagnosis and respective resection, but death at 181 months for the last recurrence

US, ultrasonography; CT, computed tomography; FNAC, fine-needle aspiration cytology; FNCLCC, grading system of French Federation Cancer Centers; N/A, not available.

## Discussion

We report a patient from China with multiple-recurrent and multi-operative giant RP DDLPS for a 15-year follow-up. She was first diagnosed in 1997, experienced four recurrences, and underwent subsequent surgeries four times. This case was rare due to the more challenging treatment involved and was, by far, the longest follow-up. The recurrence of giant RP DDLPS many times caused greater operative difficulties and a poorer prognosis. The 8-year overall survival (OS) rate is only 30% of the neoplasms with high grade III(GIII). Meanwhile, the high-grade II(GII) tumor usually causes death by recurrence or metastasis (7). When managed in our hospital in July 2010, the patient suffered from a poor medical condition during the perioperative period. The multifocal giant tumors invaded the stomach with 2800 mL of estimated blood loss, high-grade and advanced pathological presentations, and complicated surgery. However, we accomplished R0 resection and a smooth postoperative process, which would be instructive for improving the management standards of such complicated cases. Because of the fear of the disease and the pain of each operation, the patient felt miserable on account of all these recurrences. However, she felt no particular discomfort and recovered smoothly. The levels of adherence and tolerability of the patient were favorable. Moreover, there were no adverse or unanticipated events.

Soft tissue sarcomas account for less than 1% of all neoplasms (19). While RPLS is more uncommon and hardly diagnosed early, it progresses to present some manifestations such as stomachache, ventosity, palpable abdominal mass, compressed surrounding viscera, and gastrointestinal hemorrhage. Computed tomography (CT) or magnetic resonance imaging (MRI) is the primary diagnosis (20, 21). CT may frequently establish a preliminary diagnosis by indicating the location, size, consistency, and relationships with adjacent structures and can provide some proposals for surgery. For possible seeding in the needle tract and low accuracy in diagnosing RP DDLPS, we do not generally recommend *core needle* biopsy guided by CT (22, 23). However, it can be applied to preoperative radiotherapy and radiochemotherapy or target genomic therapy for unresectable tumors (24).

According to the 2013 World Health Organization (WHO) classification of soft tissue and bone tumors, RPLS is classified into four main subtypes: well-differentiated, myxoid/round cell, dedifferentiated, and pleomorphic (25). The 5-year survival rates of well-differentiated and myxoid/round cell variations are 90% and 60%–90%, respectively, with relatively better prognoses; the rates of dedifferentiated and pleomorphic varieties are 75% and 30%–50%, respectively (24). The pleomorphic subtype is most likely to metastasize distantly. A total of 83% of RP DDLPS patients experience locoregional recurrences, and 10%–15% of them develop distant metastasis postoperatively. Furthermore, 30% of recurrent tumors metastasize within the first 3 years (26). According to FNCLCC, RPLS is classified as grade I, II, and III (27). The well-differentiated and myxoid/round cell subtypes are low grade,, although the pleomorphic and dedifferentiated subtypes have higher grades with poorer outcomes (28). The histological subtype, grade, and complete surgical resection (R0) are the main prognostic factors (29). Moreover, the multifocal growth and rapid growth rate after recurrence (an average of more than 0.9 cm/month) are the influencing factors (30, 31).

Radical surgery, including invading tissues and organs, is the mainstay of treatment that can effectively cure RPLS, reduce the risk of a recurrence, and improve disease-free survival (DFS) and overall survival (OS) rates (32). Lewis reported a cohort of 500 patients with retroperitoneal soft-tissue sarcoma in a single institution. The median survival was 103 months for patients with thorough surgery vs. 18 months for those with incomplete resection (5). Zeng presented that they organized a multidisciplinary team to draw up a meticulous plan by establishing abdominal CT aortography and applying intraureteral catheterization to achieve complete resection (13). The application of adjuvant radiochemotherapy is still controversial (24). Haas reported that neoadjuvant radiotherapy might improve the local recurrence rate but with no benefit in OS (33). Postoperative radiotherapy, even if applied restrictedly, can cause some damage to the normal surrounding tissues and lead to related complications (34). The effectiveness of chemotherapy has not been clearly demonstrated, and this treatment has not been generally carried out so far; only in some isolated reports does it find a mention (35, 36).

Nevertheless, radiochemotherapy is still applied as palliative treatment to patients with unresectable or distant metastasis, which may improve the quality of life to some extent and prolong the period of survival(24). Research on target genomic therapy has become a hotspot, aiming to improve surgical outcomes in recent years in order to overcome the various limitations of radiochemotherapy. For instance, the dedifferentiated variety, aimed at the genes harboring the amplified sequences on chromosome 12(12q13–15), the antagonists or inhibitors of *MDM*2, *CDK*4, *HMGA*2, et al., and the ligand for *PPAR-γ* have been developed. However, to reduce the impact of the adverse effects and resistance of *MDM*2 inhibitors, we should pay more attention to translating research with *YEATS*4 knockdown and the genes outside the amplicon. Therefore, inhibiting *JUN*, *DDR*2, *FGFR*3, *NTRK*1, lowering or absenting *ZIC-*1, and recovering the normal expressions of *RB*1 and *CEBPA* are all research approaches(37).

However, our research did not involve any systematic and comprehensive study of the number of case limits, the patient's complicated illness, and managerial criteria. Nevertheless, it would provide some scientific evidence and support for the standardized management of such cases in our tertiary medical center or at WHO. In the future, we hope to establish multicenter databases, formulate standardized operative procedures, study target genomic therapy, and, if possible, normalize diagnostic and therapeutic programs.

## Conclusion

An RPLS measuring greater than 30 cm in diameter is extremely rare and is considered a “giant liposarcoma.” The neoplasms tend to multiple-recur locally. Approximately 10%–15% of patients with RP DDLPS may develop distant metastasis, with rare chances of long-term survival. The primary diagnostic tools are CT and MRI scans. Complete surgical resection with infiltrated structures is the cornerstone of cancer and its locoregional relapses. The prognosis of RPLS is associated with pathological subtype, grade, and radical surgery (R0); hence, genomic treatment has been receiving a lot of attention recently. Given the recurrent tendency and genomic characteristics of RPLS, we may hope to establish genetic screening of primary and recurrent patients to prevent recidive by target therapy and consequently improve the prognosis.

## Data Availability

The original contributions presented in the study are included in the article/supplementary files, further inquiries can be directed to the corresponding author/s.

## References

[1] Mettlin C Priore R Rao U. Results of the national soft tissue sarcoma registry. J Surg Oncol. (1982) 19:224–7. 10.1002/jso.2930190410 7078175

[2] Herrera-Gómez A Ortega-Gutiérrez C Betancourt AM Luna-Ortiz K. Giant retroperitoneal liposarcoma. World J Surg Oncol. (2008) 6:115. 10.1186/1477-7819-6-115 18976464 PMC2644689

[3] Mack TM. Sarcomas and other malignancies of soft tissue, retroperitoneum, peritoneum, pleura, heart, mediastinum, and spleen. Cancer. (1995) 75:211–44. 10.1002/1097-0142(19950101)75:1+<211::AID-CNCR2820751309>3.0.CO;2-X 8000998

[4] Mendenhall WM Zlotecki RA Hochwald SN Hemming AW Grobmyer SR Cance WG. Retroperitoneal soft tissue sarcoma. Cancer. (2005) 104:669–75. 10.1002/cncr.21264 16003776

[5] Lewis JJ Leung D Woodruff JM Brennan MF. Retroperitoneal soft-tissue sarcoma: analysis of 500 patients treated and followed at a single institution. Ann Surg. (1998) 228:355–65. 10.1097/00000658-199809000-00008 9742918 PMC1191491

[6] Bagaria SP Gabriel E Mann GN. Multiply recurrent retroperitoneal liposarcoma. J Surg Oncol. (2018) 117:62–8. 10.1002/jso.24929 29266232

[7] Gronchi A Strauss DC Miceli R Bonvalot S Swallow CJ Hohenberger P Variability in patterns of recurrence after resection of primary retroperitoneal sarcoma (RPS): a report on 1007 patients from the multi-institutional collaborative RPS working group. Ann Surg. (2016) 263:1002–9. 10.1097/SLA.0000000000001447 26727100

[8] Ianoşi G Neagoe D Buteică E Ianoşi S Drighiciu C Stănoiu B Giant retroperitoneal sarcomas. Rom J Morphol Embryol. (2007) 48:303–8. 17914501

[9] Han HH Choi KH Kim DS Jeong WJ Yang SC Jang SJ Retroperitoneal giant liposarcoma. Korean J Urol. (2010) 51:579–82. 10.4111/kju.2010.51.8.579 20733966 PMC2924564

[10] Hashimoto Y Hatakeyama S Tachiwada T Yoneyama T Koie T Kamimura N Surgical treatment of a giant liposarcoma in a Japanese man. Adv Urol. (2010) 2010:943073. 10.1155/2010/943073 21197426 PMC3010627

[11] Caizzone A Saladino E Fleres F Paviglianiti C Iaropoli F Mazzeo C Giant retroperitoneal liposarcoma: case report and review of the literature. Int J Surg Case Rep. (2015) 9:23–6. 10.1016/j.ijscr.2015.02.019 25722109 PMC4392328

[12] Oh SD Oh SJ Suh BJ Shin JY Oh CK Park JK A giant retroperitoneal liposarcoma encasing the entire left kidney and adherent to adjacent structures: a case report. Case Rep Oncol. (2016) 9:368–72. 10.1159/000447488 27462239 PMC4939687

[13] Zeng XY Liu WZ Wu XL Gao JB Zhang P Shuai XM Clinicopathological characteristics and experience in the treatment of giant retroperitoneal liposarcoma: a case report and review of the literature. Cancer Biol Ther. (2017) 18:660–5. 10.1080/15384047.2017.1345388 28758856 PMC5663413

[14] Hazen B Cocieru A. Giant retroperitoneal sarcoma. J Gastrointest Surg. (2017) 21:602–3. 10.1007/s11605-016-3258-0 27613734

[15] Herzberg J Niehaus K Holl-Ulrich K Honarpisheh H Guraya SY Strate T. Giant retroperitoneal liposarcoma: a case report and literature review. J Taibah Univ Med Sci. (2019) 14:466–71. 10.1016/j.jtumed.2019.08.005 31728146 PMC6839011

[16] Xu C Ma Z Zhang H Yu J Chen S. Giant retroperitoneal liposarcoma with a maximum diameter of 37 cm: a case report and review of the literature. Ann Transl Med. (2020) 8:1248. 10.21037/atm-20-1714 33178780 PMC7607090

[17] El-Helou E Alimoradi M Sabra H Naccour J Haddad MM Bitar H. Recurrent giant retroperitoneal liposarcoma with 10 years follow up. Case report and review of literature. Int J Surg Case Rep. (2020) 75:504–12. 10.1016/j.ijscr.2020.09.143 33076205 PMC7530305

[18] Kuperus JM Steensma MR Khachaturov V Lane BR. Surgical management of a large retroperitoneal liposarcoma: a case study. Urol Case Rep. (2020) 34:101502. 10.1016/j.eucr.2020.101502 33318934 PMC7726650

[19] WHO Classification of Tumours Editorial Board. WHO classification of tumours of soft tissue and bone. 5th ed. Lyon, France: IARC Press (2020).

[20] Yu JSE Colborne S Hughes CS Morin GB Nielsen TO. The FUS-DDIT3 interactome in myxoid liposarcoma. Neoplasia. (2019) 21:740–51. 10.1016/j.neo.2019.05.004 31220736 PMC6584455

[21] Abaricia S Hirbe AC. Diagnosis and treatment of myxoid liposarcomas: histology matters. Curr Treat Options Oncol. (2018) 19:64. 10.1007/s11864-018-0590-5 30362022

[22] Clark MA Thomas JM. Portsite recurrence after laparoscopy for staging of retroperitoneal sarcoma. Surg Laparosc Endosc Percutan Tech. (2003) 13:290–1. 10.1097/00129689-200308000-00015 12960797

[23] Ikoma N Torres KE Somaiah N Hunt KK Cormier JN Tseng W Accuracy of preoperative percutaneous biopsy for the diagnosis of retroperitoneal liposarcoma subtypes. Ann. Surg. Oncol. (2015) 22:1068–72. 10.1245/s10434-014-4210-8 25354575 PMC4520392

[24] Vijay A Ram L. Retroperitoneal liposarcoma: a comprehensive review. Am J Clin Oncol. (2015) 38:213–9. 10.1097/COC.0b013e31829b5667 24136142

[25] Dei Tos AP. Liposarcomas: diagnostic pitfalls and new insights. Histopathology. (2014) 64:38–52. 10.1111/his.12311 24118009

[26] Singer S Antonescu CR Riedel E Brennan MF. Histologic subtype andmargin of resection predictpattern of recurrence and survival for retroperitoneal liposarcoma. Ann Surg. (2003) 238:358–71. 10.1097/01.sla.0000086542.11899.38 14501502 PMC1422708

[27] Matthyssens LE Creytens D Ceelen WP. Retroperitoneal liposarcoma: current insights in diagnosis and treatment. Front Surg. (2015) 2:4. 10.3389/fsurg.2015.00004 25713799 PMC4322543

[28] Fletcher C Unni K Mertens F. Pathology and genetics of tumors of soft tissue and bone. In: Kleihues P, editors. World health organization classifification of tumors. Lyon: International Agency for Research on Cancer Press (2002). p. 427.

[29] van Houdt WJ Zaidi S Messiou C Thway K Strauss DC Jones RL. Treatment of retroperitoneal sarcoma: current standards and new developments. Curr Opin Oncol. (2017) 29:260–7. 10.1097/CCO.0000000000000377 28509807

[30] Anaya DA Lahat G Liu J Xing Y Cormier JN Pisters PW Multifocality in retroperitoneal sarcoma: a prognostic factor critical to surgical decisionmaking. Ann Surg. (2009) 249:137–42. 10.1097/SLA.0b013e3181928f2f 19106689

[31] Park JO Qin LX Prete FP Antonescu C Brennan MF Singer S. Predicting outcome by growth rate of locally recurrent retroperitoneal liposarcoma: the one centimeter per month rule. Ann Surg. (2009) 250:977–82. 10.1097/SLA.0b013e3181b2468b 19953716 PMC3248745

[32] Gronchi A Miceli R Shurell E Eilber FC Eilber FR Anaya DA Outcome prediction in primary resected retroperitoneal soft tissue sarcoma: histology-specific overall survival and disease-free survival nomograms built on major sarcoma center data sets. J Clin Oncol. (2013) 31:1649–55. 10.1200/JCO.2012.44.3747 23530096

[33] Haas RLM Bonvalot S Miceli R Strauss DC Swallow CJ Hohenberger P Radiotherapy for retroperitoneal liposarcoma: a report from the transatlantic retroperitoneal sarcoma working group. Cancer. (2019) 125:1290–300. 10.1002/cncr.31927 30602058 PMC6590287

[34] Nussbaum DP Speicher PJ Gulack BC Ganapathi AM Englum BR Kirsch DG Long-term oncologic outcomes after neoadjuvant radiation therapy for retroperitoneal sarcomas. Ann Surg. (2015) 262:163–70. 10.1097/SLA.0000000000000840 25185464 PMC4345136

[35] Bachmann R Eckert F Gelfert D Strohäker J Beltzer C Ladurner R. Perioperative strategy and outcome in giant retroperitoneal dedifferentiated liposarcoma-results of a retrospective cohort study. World J Surg Oncol. (2020) 18:296. 10.1186/s12957-020-02069-2 33183309 PMC7664077

[36] Yokoyama Y Nishida Y Ikuta K Nagino M. A case of retroperitoneal dedifferentiated liposarcoma successfully treated by neoadjuvant chemotherapy and subsequent surgery. Surg Case Rep. (2020) 6:105. 10.1186/s40792-020-00865-2 32448975 PMC7246274

[37] Tyler R Wanigasooriya K Taniere P Almond M Ford S Desai A A review of retroperitoneal liposarcoma genomics. Cancer Treat Rev. (2020) 86:102013. 10.1016/j.ctrv.2020.102013 32278233

